# Impact of Comorbidities Among Medicaid Enrollees With Chronic Obstructive Pulmonary Disease, United States, 2009

**DOI:** 10.5888/pcd14.160333

**Published:** 2017-04-13

**Authors:** Gloria Westney, Marilyn G. Foreman, Junjun Xu, Marshaleen Henriques King, Eric Flenaugh, George Rust

**Affiliations:** 1 Pulmonary and Critical Care Division, Morehouse School of Medicine, Atlanta, Georgia; 2 National Center for Primary Care, Morehouse School of Medicine, Atlanta, Georgia

## Abstract

**Introduction:**

Multimorbidity, the presence of 2 or more chronic conditions, frequently affects people with chronic obstructive pulmonary disease (COPD). Many have high-cost, highly complex conditions that have a substantial impact on state Medicaid programs. We quantified the cost of Medicaid-insured patients with COPD co-diagnosed with other chronic disorders.

**Methods:**

We used nationally representative Medicaid claims data to analyze the impact of comorbidities (other chronic conditions) on the disease burden, emergency department (ED) use, hospitalizations, and total health care costs among 291,978 adult COPD patients. We measured the prevalence of common conditions and their influence on COPD-related and non–COPD-related resource use by using the Elixhauser Comorbidity Index. Elixhauser comorbidity counts were clustered from 0 to 7 or more. We performed multivariable logistic regression to determine the odds of ED visits by Elixhauser scores adjusting for age, sex, race/ethnicity, and residence.

**Results:**

Acute care, hospital bed days, and total Medicaid-reimbursed costs increased as the number of comorbidities increased. ED visits unrelated to COPD were more common than visits for COPD, especially in patients self-identified as black or African American (designated black). Hypertension, diabetes, affective disorders, hyperlipidemia, and asthma were the most prevalent comorbid disorders. Substance abuse, congestive heart failure, and asthma were commonly associated with ED visits for COPD. Female sex was associated with COPD-related and non–COPD-related ED visits.

**Conclusion:**

Comorbidities markedly increased health services use among people with COPD insured with Medicaid, although ED visits in this study were predominantly unrelated to COPD. Achieving excellence in clinical practice with optimal clinical and economic outcomes requires a whole-person approach to the patient and a multidisciplinary health care team.

## Introduction

Chronic obstructive pulmonary disease (COPD) results primarily from tobacco smoking. COPD is the third leading cause of death in the United States and is notable for recent increases in COPD-related deaths in middle-aged black and white women (1,2). COPD is a systemic disorder in which extrapulmonary manifestations (ie, cachexia and osteoporosis) may be prominent. Because of smoking, other risk factors, and the systemic effects of the disease, people with COPD often have complex clusters of chronic conditions. The medical complexity of these multiple conditions may also be compounded by mental health or substance use disorders and by poverty or other social factors.

Previous data from the Medicare population showed that multiple chronic conditions, (comorbidities) are associated with adverse outcomes, forming the basis for risk stratification in population health management programs (3). Before the Affordable Care Act, and in states that have not participated in Medicaid expansion, the segment of adult Medicaid recipients not covered by Medicare who have disability as their basis for Medicaid eligibility often accounts for a large portion of Medicaid spending in each state. No large study has documented the burden of disease, cost, or adverse event rates for COPD as a component of people with various clusters of chronic conditions in the Medicaid population. Therefore, the purpose of this study was to measure the impact of comorbidities on the disease burden, disease outcomes, and costs associated with COPD in people insured by Medicaid.

## Methods

### Study population

We used a retrospective cohort design to analyze the effects of comorbidities on health care outcomes among people with COPD. The study population was drawn from the 2009 Medicaid Analytic eXtract (MAX) file obtained from Centers for Medicare and Medicaid Services. We extracted data for adults aged 18 to 64 years from 28 states (Alabama, Arizona, Arkansas, California, Colorado, Connecticut, Florida, Georgia, Illinois, Indiana, Louisiana, Maryland, Massachusetts, Michigan, Mississippi, Missouri, New Jersey, New Mexico, New York, North Carolina, Ohio, Oklahoma, Pennsylvania, South Carolina, Tennessee, Texas, Virginia, Washington) and the District of Columbia, which accounted for 80% of all Medicaid enrollees in the United States and 90% of black Medicaid enrollees. By using *International Classification of Diseases, 9^th^ Revision* diagnostic codes 491.0, 491.1, 491.2, 491.8, 492.xx, 493.2, 494.xx, 496.xx, we identified patients with COPD for this study if they had 1 or more billed claims from the inpatient file or at least 2 billed claims from the outpatient file (4). Eligible patients must have been enrolled in Medicaid for 12 months in 2009. We excluded patients with a Medicaid health maintenance organization plan and patients simultaneously eligible for both Medicare and Medicaid because necessary encounter data could not be fully accessed from our data set.

### Measures

The outcomes in our study were emergency department (ED) visits, hospital bed days, and total Medicaid costs. ED visits were identified by any of the following codes for professional claims: 1) place of service code 23 (ED–hospital); 2) hospital revenue codes 450 to 459; or 3) the Centers for Medicare and Medicaid Current Procedural Terminology Codes 99,281 to 99,285. ED visits were classified as COPD-related if the primary diagnostic code was COPD; otherwise, ED visits were considered other-cause (non–COPD-related). Hospital bed days and total Medicaid costs were obtained from the personal summary file.

We calculated comorbidities, the main predictor in our study, by using the Elixhauser Comorbidity Index, a validated approach that summarizes disease burden and predicts risks by using administrative claims data (5). We categorized the comorbidity index into 5 groups (0, 1 or 2, 3 or 4, 5 or 6, ≥7) and assigned each patient to one of these groups.

Covariates were age, sex, race/ethnicity, and rural or urban residence. We analyzed age in 3 groups (18–29 y, 30–44 y, and 45–64 y) and race/ethnicity in 5 categories (white, black, Hispanic, Asian, and other). Rural–urban designation was acquired from the Area Health Resources File (6). We merged Area Health Resources File data to MAX data with matching county-level Federal Information Processing Standards codes. Based on 2012–2013 rural/urban continuum codes from the US Department of Agriculture’s Economic Research Services, counties were classified as large metropolitan (population >1 million), small metropolitan (population from 250,000 to 1 million), and rural (population <250,000).

### Analysis

We conducted descriptive statistics analysis to characterize our sample by comorbidity index and demographics. We performed 1-way analysis of variance tests to estimate mean differences in number of ED visit, hospital bed days, and total Medicaid costs across predictor and covariate subgroups. Multivariate logistic regression models were used to evaluate the odds of ED visits (primary and all-cause) and comorbidities, mutually adjusting for age, sex, race/ethnicity, and residence. The threshold for statistical significance was set at *P* ≤ .05. All analyses were performed with SAS version 9.3 (SAS Institute, Inc).

The study was conducted in accordance with the Amended Declaration of Helsinki and approved by the Morehouse School of Medicine Biomedical Institutional Review Board (approval no. 465968–3).

## Results

Sixty-two percent of the 291,978 people in our study were female, 67% were white, 85% were aged 45 to 64 years, and 38% were residents of large metropolitan areas (Table 1). Comorbidities were distributed as follows: no additional Elixhauser-defined comorbidities, 24%; 1 or 2 comorbidities, 38%; 3 or 4 comorbidities; 21%; 5 or 6 comorbidities, 10%; and 7 or more comorbidities, 6% (percentages do not total 100 because of rounding). ED visits were COPD-related in 13% of participants, and 60% had ED visits for other reasons. The average hospital length of stay was 5.6 days (standard deviation 16.5), and total Medicaid costs per person were $23,825 (median, $10,803) (Table 2).

**Table 1:** Demographic and Health-Related Characteristics of Medicaid Enrollees With Chronic Obstructive Pulmonary Disease (N = 291,978), United States, 2009

Characteristic	No. (%)
**Age, y**	
18–29	6,001 (2.1)
30–44	38,345 (13.1)
45–64	247,632 (84.8)
**Race/ethnicity**	
White	194,295 (66.5)
Black	58,972 (20.2)
Hispanic	10,806 (3.7)
Asian	1,140 (0.4)
Other	26,765 (9.2)
**Sex**	
Female	181,260 (62.1)
Male	110,718 (37.9)
**Residence^a^**	
Large metropolitan area	110,908 (38.0)
Small metropolitan area	93,061 (31.9)
Rural	88,009 (30.1)
**Elixhauser Comorbidity Index**	
0	70,133 (24.0)
1 or 2	112,195 (38.4)
3 or 4	61,584 (21.1)
5 or 6	29,728 (10.2)
≥7	18,338 (6.3)
**COPD-related emergency department visits**	
Yes	37,520 (12.9)
No	254,458 (87.2)
**Non–COPD-related emergency department visits**	
Yes	175,704 (60.2)
No	116,274 (39.8)

Abbreviation: COPD, chronic obstructive pulmonary disease.

a Based on 2012–2013 rural/urban continuum codes from the US Department of Agriculture’s Economic Research Services; counties were classified as large metropolitan (population >1 million), small metropolitan (population from 250,000 to 1 million), and rural (population <250,000).

**Table 2:** Characteristics of Hospital Visits, Medicaid Enrollees With Chronic Obstructive Pulmonary Disease (COPD), United States, 2009

Type of Visit	Mean (SD)	Median (Q1–Q3)
Emergency department visits, COPD-related	0.2 (0.9)	0
Emergency department visits, non–COPD–related	2.6 (5.4)	1 (0–3)
Hospital bed days	5.6 (16.4)	0 (0–5)
Total Medicaid cost, $	23,825 (38,639)	10,803 (2,881–8,434)

Abbreviations: Q, quartile; SD, standard deviation.

COPD-related ED visits were most common among people aged 45 to 64 years, whites, women, and rural residents (*P* < .001) (Table 3). Rural Medicaid enrollees and women were most likely to have both COPD-related ED visits and ED visits for all other-causes (*P* < .001). The highest prevalence of non–COPD-related ED visits was among Medicaid enrollees aged 18 to 29 years and blacks (*P* < .001). Men, people aged 18 to 29 years, and those from large metropolitan areas had significantly longer hospital bed days and higher total Medicaid cost (*P* < .001) than other enrollees with COPD. Black enrollees had longer hospital bed days than other races/ethnicities, but Asian enrollees’ total Medicaid costs were on average the highest (*P* < .001). We saw a dose-dependent relationship between each increase in the Elixhauser Comorbidity Index and ED visits, hospital bed days, and total Medicaid costs (Figure). 

**Table 3:** Average Emergency Department (ED) Visits, Hospital Bed Days, and Total Medicaid Costs, Medicaid Enrollees With Chronic Obstructive Pulmonary Disease (COPD), United States, 2009

Characteristic	COPD-Related ED Visit		All Other-Cause ED Visit		COPD-Related ED Visit		All Other Cause ED Visits		Hospital Bed days		Total Medicaid Cost, $	
	%	*P* Value	%	*P* Value	Mean (SD)	*P* Value	Mean (SD)	*P* Value	Mean (SD)	*P* Value	Mean (SD)	*P* Value
**Total**	12.9	NA	60.2	NA	0.2 (0.9)	NA	2.6 (5.4)	NA	5.6 (16.4)	NA	23,825 (38,640)	NA
**Age, y**												
18–29	5.3	.001	70.9	.001	0.1 (0.6)	.001	4.2 (7.9)	.001	10.1 (25.5)	.001	47,553 (77,259)	.001
30–44	10.8		69.6		0.2 (0.7)		3.8 (7.2)		6.1 (18.1)		23,903 (41,018)	
45–64	13.4		58.5		0.2 (0.9)		2.3 (4.9)		5.5 (15.8)		23,238 (36,623)	
**Race/ethnicity**												
White	13.2	.001	58.9	.001	0.2 (0.9)	.001	2.4 (5.0)	.001	4.7 (13.8)	.001	21,547 (34,837)	.001
Black	12.7		66.0		0.2 (1.0)		3.2 (6.5)		8.3 (22.7)		28,550 (44,837)	
Hispanic	7.7		53.0		0.1 (0.5)		2.2 (5.0)		6.4 (16.4)		28,490 (43,637)	
Asian	8.1		49.7		0.1 (0.6)		1.7 (3.5)		6.1 (17.4)		29,392 (49,508)	
Other	12.9		60.1		0.2 (0.9)		2.5 (5.1)		6.0 (16.2)		27,831 (45,408)	
**Sex**												
Female	13.3	.001	61.6	.001	0.2 (0.8)	.92	2.6 (5.0)	.13	5.2 (15.3)	.001	22,267 (35,719)	.001
Male	12.1		57.9		0.2 (1.0)		2.5 (5.9)		6.3 (18.0)		26,375 (42,873)	
**Residence^a^**												
Large metropolitan area	12.3	.001	59.5	.001	0.2 (1.0)	.36	2.8 (6.0)	.001	7.7 (20.8)	.001	30,812 (48,151)	.001
Small metropolitan area	13.1		60.1		0.2 (0.8)		2.5 (5.3)		4.8 (13.4)		20,900 (33,258)	
Rural	13.3		61.1		0.2 (0.8)		2.3 (4.5)		4.0 (12.2)		18,112 (27,543)	
**Elixhauser Comorbidity Index**												
0	6.7	.001	34.9	.001	0.1 (0.4)	.001	0.8 (2.5)	.001	0.4 (3.2)	.001	11,934 (24,640)	.001
1 or 2	11.6		55.8		0.2 (0.6)		1.8 (3.9)		2.2 (7.5)		16,455 (25,641)	
3 or 4	16.2		75.3		0.3 (1.0)		3.3 (5.4)		7.1 (13.8)		27,575 (35,868)	
5 or 6	19.1		84.6		0.4 (1.5)		4.7 (7.3)		13.6 (22.6)		41,497 (49,226)	
≥7	22.9		93.1		0.5 (1.7)		7.8 (10.3)		29.1 (39.9)		73,152 (72,931)	

Abbreviations: NA, not applicable; SD, standard deviation.

a Based on 2012–2013 rural/urban continuum codes from the US Department of Agriculture’s Economic Research Services; counties were classified as large metropolitan (population >1 million), small metropolitan (population from 250,000 to 1 million), and rural (population <250,000).

**Figure:**
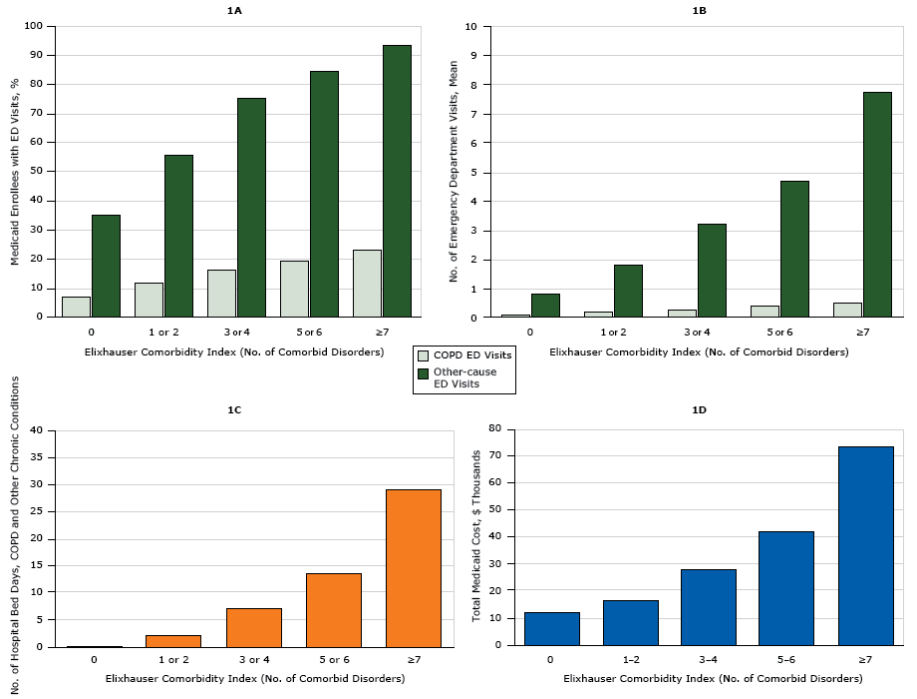
Use of health care resources by adults aged 18 to 64 years with chronic obstructive pulmonary disease (COPD) and 1 or more additional chronic disorders enrolled in Medicaid in 2009, based on 5 clusters of the Elixhauser Comorbidity Index. 1A shows the percentage of all emergency department (ED) visits in 2009 made for COPD and for other chronic disorders. 1B shows the number of ED visits for COPD and for other causes. 1C shows the average number of hospital bed days for COPD and other causes. 1D shows total Medicaid costs for hospital treatment of COPD and other chronic disorders. **Table:** 

Elixhauser Comorbidity Index (No. of Comorbid Disorders)	1A. Prevalence of ED Visits, %		1B. No. of ED Visits		1C. Hospital Bed Days for COPD and Other Chronic Conditions	1D. Total Medicaid Cost, $
	COPD-Related	Other Causes	COPD-Related	COPD-Related		
0	6.7	34.9	0.086	0.826	0.418	11,934
1 or 2	11.6	55.8	0.173	1.816	2.163	16,455
3 or 4	16.2	75.3	0.284	3.263	7.118	27,575
5 or 6	19.1	84.6	0.390	4.738	13.574	41,497
≥7	22.9	93.1	0.525	7.751	29.091	73,152

We calculated associations between ED visits and covariates on the basis of logistic regression models (Table 4). Among people with COPD, having 1 or more chronic conditions was a significant predictor of both COPD-related ED visits and all other-cause ED visits. The strongest association was detected among people with 7 or more comorbidities and COPD-related ED visits (adjusted odds ratio [AOR] = 4.26; 95% CI, 4.07–4.46) and other ED visits (AOR = 26.58; 95% CI, 25.05–28.21). Compared with enrollees aged 45 to 64 years, both younger age groups (18–29 y and 30–44 y) were less likely to have COPD-related ED visits but significantly more likely to have all other-cause ED visits. Whites were more likely to have COPD-related ED visits than all other racial/ethnic groups. Being Hispanic (OR = 0.78; 95% CI, 0.75–0.81) and of Asian race/ethnicity (OR = 0.76; 95% CI, 0.67–0.86) was associated with lower rates of all other-cause ED visits. Female sex was a significant predictor of both types of ED visits. Residence in metropolitan areas, both large and small, was associated with fewer ED visits, COPD-related and non–COPD-related.

**Table 4:** Odds of Emergency Department Visits Among Medicaid Enrollees with Chronic Obstructive Pulmonary Disease (COPD), by Demographic and Health Characteristics, United States, 2009

Characteristic	COPD-Related Emergency Department Visits				All Other Emergency Department Visits			
	Crude OR (95% CI)	*P* Value	Adjusted OR (95% CI)	*P* Value	Crude OR (95% CI)	*P* Value	Adjusted OR (95% CI)	*P *Value
**Age, y**								
45–64	1 [Reference]							
18–29	0.37 (0.33–0.41)	<.001	0.41 (0.37–0.46)	<.001	1.73 (1.64–1.83)	<.001	2.32 (2.19–2.47)	<.001
30–44	0.79 (0.76–0.81)	<.001	0.81 (0.78–0.84)	<.001	1.63 (1.59–1.67)	<.001	1.89 (1.84–1.94)	<.001
**Race/ethnicity**								
White	1 [Reference]							
Black	0.95 (0.93–0.98)	<.001	0.85 (0.83–0.88)	<.001	1.35 (1.33–1.38)	<.001	1.12 (1.10–1.15)	<.001
Hispanic	0.55 (0.51–0.59)	<.001	0.57 (0.53–0.62)	<.001	0.79 (0.76–0.82)	<.001	0.78 (0.75–0.81)	<.001
Asian	0.58 (0.47–0.71)	<.001	0.61 (0.50–0.76)	<.001	0.69 (0.62–0.78)	<.001	0.76 (0.67–0.86)	<.001
Other	0.97 (0.94–1.01)	.14	0.95 (0.91–0.98)	.005	1.05 (1.02–1.08)	<.001	1.03 (1.00–1.06)	.07
**Sex**								
Male	1 [Reference]							
Female	1.11 (1.09–1.14)	<.001	1.10 (1.08–1.13)	<.001	1.17 (1.15–1.18)		1.18 (1.16–1.20)	<.001
**Residence^a^**								
Rural	1 [Reference]							
Large metropolitan area	0.91 (0.89–0.94)	<.001	0.91 (0.89–0.94)	<.001	0.93 (0.92–0.95)	<.001	0.83 (0.82–0.85)	<.001
Small metropolitan area	0.98 (0.95–1.01)	.12	0.97 (0.95–1.00)	.04	0.96 (0.94–0.98)	<.001	0.92 (0.90–0.93)	
**Elixhauser Comorbidity Index**								
0	1 [Reference]							
1 or 2	1.84 (1.78–1.90)	<.001	1.84 (1.78–1.91)	<.001	2.36 (2.31–2.40)	<.001	2.37 (2.33–2.42)	<.001
3 or 4	2.71 (2.61–2.81)	<.001	2.72 (2.62–2.82)	<.001	5.68 (5.54–5.81)	<.001	5.88 (5.74–6.02)	<.001
5 or 6	3.31 3.17–3.45)	<.001	3.34 (3.20–3.49)	<.001	10.20 (9.85–0.56)	<.001	10.68 (10.31–11.07)	<.001
≥7	4.15 (3.97–4.35)	<.001	4.26 (4.07–4.46)	<.001	25.10 (23.6–6.63)	<.001	26.58 (25.05−28.21)	<.001

Abbreviations: CI, confidence interval; OR, odds ratio.

a Based on 2012–2013 rural/urban continuum codes from the US Department of Agriculture’s Economic Research Services; counties were classified as large metropolitan (population >1 million), small metropolitan (population from 250,000 to 1 million), and rural (population <250,000).

In addition to applying the Elixhauser Comorbidity Index, we also measured the frequency of common chronic conditions. Among 291,978 people with COPD, the 5 leading chronic conditions were hypertension (46%), diabetes (31%), affective disorders (27%), hyperlipidemia (20%), and asthma (18%). The highest prevalence of COPD-related ED visits occurred among people whose comorbid conditions were alcohol abuse (20%), congestive heart failure (20%), and asthma (20%). Prevalence of all other-cause ED visits was associated with patients having comorbid alcohol abuse (89%), sickle cell disease (82%), and liver failure (80%).

## Discussion

This study documented the high prevalence of comorbid conditions among people with COPD enrolled in Medicaid in 28 states and the District of Columbia. We also documented the high frequency of ED visits among people with COPD. We quantified the extent to which ED visits, hospital admissions, and total Medicaid costs rose incrementally as the number of co-existent medical conditions increased. In addition, findings indicated sex and racial/ethnic differences in adjusted odds of all-cause emergency department visits, with higher odds of an ED visit for women than for men and for blacks with COPD than for whites with COPD and lower rates among Hispanic or Latino and Asian patients than among other racial/ethnic groups.

The fiscal impact of COPD on health care costs is predicted to continue to increase through 2020 (7). Even in a population of people with COPD with national health insurance, people with COPD disproportionately use health services (8). Other studies have documented high levels of health care use and costs in patients with COPD (9–12).

Comorbidities occur more commonly among people with COPD than in the general population and are associated with reduced quality of life and increased health care costs (13,14). Our study is consistent with other reports documenting the burden of comorbid disorders among people with COPD in addition to documenting racial differences in outcomes (15–17). Singh and Yu (18) used the 2009–2012 National Emergency Department sample to analyze predictors of emergency department visits for COPD, emphasizing outcomes, costs, hospitalizations, and discharges. In that study, approximately 63% of participants were Medicare recipients. The study reported that costs for COPD ED visits over time increased and that the comorbidities associated with increased risk for hospitalization were hypertension, diabetes, coronary artery disease, heart failure, and hyperlipidemia. The Singh and Yu study was designed to determine the effect of individual comorbidities rather than the aggregated effects of multiple chronic conditions. In their report, older age, higher income, metropolitan residence, female sex, residence in the northeast United States, hypertension, heart failure, coronary artery disease, hyperlipidemia, diabetes, renal failure, and osteoarthritis were associated with higher odds of hospitalization. Gershon et al (19) confirmed increased health resource use in patients with COPD, compared with similar patients without COPD, and also reported higher adjusted relative rates of most chronic disorders in younger people with COPD (aged 35–64 y) than in older people without COPD. In this longitudinal study, younger people with COPD had higher rates of ambulatory care visits, ED visits, and hospitalizations for various chronic conditions (all-cause, cardiovascular disease, diabetes, low trauma fractures, lung cancer, musculoskeletal disease, non-lung cancer, and psychiatric disorders) with the exception of ambulatory and ED visits for lower respiratory tract infection. This study further substantiates the impact and importance of other chronic disorders in association with COPD. The higher relative rates in younger COPD patients suggest that COPD may confer enhanced susceptibility for certain other diseases. Additional work has been done to identify conceivable or novel biologic and molecular pathways linking COPD and 16 chronic diseases (20). Systemic inflammation has been demonstrated in COPD patients with concurrent hypertension, diabetes, or cardiovascular disease. Risk factors common to COPD and other disorders include tobacco smoking, inactivity, and aging.

The current focus on value-driven health care emphasizes all-cause, whole-person outcomes rather than disease-specific outcomes. The impact of COPD and comorbidities is bidirectional. For instance, congestive heart failure (CHF) was associated with COPD-specific ED visits in our data and is a common reason for hospitalization in the United States. In a registry of more than 100,000 adult subjects with decompensated CHF, COPD was the fourth most common comorbidity (31%), only trailing hypertension (73%), coronary artery disease (57%), and diabetes (44%) (6). Analyses from ADHERE (Acute Decompensated Heart Failure National Registry) demonstrated significant variation across hospitals in compliance with key quality indicators for CHF, but adherence was greater in hospitals where comorbidities were prevalent among patients (21).

Given that comorbidities are such a driver of whole-person outcomes, we need to move beyond disease-specific guidelines to clinical guidelines for people with COPD with multiple chronic conditions. National and international respiratory societies have been grappling with the inclusion of comorbidities in COPD guidelines (22). Additional research has been recommended into the relationship between COPD and CHF or coronary artery disease with delineation of optimal treatment. Studies are also needed to address the optimal treatment of anxiety and depression in COPD patients.

Disease-specific care, commonly delivered by subspecialists, may not be optimal for people with multiple comorbidities (23). For example, what are the appropriate clinical guidelines for people with chronic lung disease, CHF, and chronic renal insufficiency? Clinical strategies must address the physical and pathophysiologic interactions of multiple chronic conditions in addition to the behavioral health and social characteristics of these patients. In a study of patients with concomitant type-2 diabetes, CHF, COPD, and chronic renal insufficiency, Bos-Touwen et al (24) found 9 explanatory determinants of levels of patient activation for self-management, all of which were “disease transcending” (ie, not COPD-specific). Aside from personal characteristics (age, body mass index, and educational status), and physical health status, the remainder were mental health (depression) or psychosocial factors (financial distress, illness perception, and social support). The prevalence of comorbid depression and alcohol use disorders among COPD patients in the Medicaid population suggests a need for clinical trials of team-based interventions (including mental health, social services, and generalist clinicians) to balance the disease focus of pulmonary, cardiology, and other subspecialty physicians.

Population health management programs have evolved from disease management to integrated care and care management across a continuum of chronic conditions. Key elements of successful programs include care coordination, patient self-management education, and health information systems to monitor adherence and outcomes, although there is wide variation in the extent to which such programs focus on COPD self-management and care coordination versus focusing on multiple chronic conditions and health outcomes management. In 2012, a report from the American Thoracic Society stated that “the integration of medical care for COPD is still in its infancy” (25), and noted that its implementation would require a significant paradigm shift. A meta-analysis of controlled trials on such interventions demonstrated significant outcomes benefits, including improvements in quality-of-life scores, 6-minute walking distance, pulmonary function, hospital admissions, and hospital bed days (26).

More extensive research is needed. The Multiple Chronic Conditions Research Network, funded by the Agency for Healthcare Research and Quality, recently offered 3 recommendations for future research: “1) include person-centered and person-driven measures and outcomes, 2) consider the person in the context of their relationships and community, and 3) include mental healthcare as an essential part of overall healthcare” (27).

Our research is subject to the usual limitations of observational studies that use claims data. For example, we did not have access to clinical records or to spirometric confirmation of COPD, but this approach to claims-based case identification has been validated and used in other reports (28,29). We also included people with a diagnosis of COPD beginning at age 18 and excluded those with dual-eligible Medicare coverage, including most patients older than 65, so our subjects skewed toward middle-aged adults who were disabled enough (not just from COPD) to qualify for Medicaid coverage. Tobacco-use–related obstructive lung disease is usually a disorder of older people, but younger patients and earlier age of onset have clinical relevance (30). Therefore, our findings are primarily generalizable to the adult, nonelderly Medicaid population and may be of interest to state Medicaid directors and clinicians serving this population. Medicaid managed care programs do not always capture encounter-level data accurately, so we excluded those enrolled in capitated managed care plans. Our findings should be confirmed independently for capitated Medicaid enrollees. We also did not attempt to measure socioeconomic characteristics, appropriateness of treatment, or polypharmacy independent of comorbidity.

This study has implications for the clinical management of patients with COPD. The primary care practitioner caring for COPD patients with comorbidities should manage multiple medical and behavioral health conditions concurrently and may need to coordinate care recommendations from multiple subspecialists. Multidisciplinary, collaborative care teams may be needed to provide optimal care for patients with multiple conditions.

Medicaid-enrolled COPD subjects have increasing rates of adverse clinical outcomes and increased costs associated with increasing prevalence of multiple chronic disorders. More research is needed to identify effective clinical guidelines for people with multiple chronic conditions, the most effective practice models, and interdisciplinary, team-based approaches to achieving optimal outcomes for patients with high levels of clinical and social complexity.
